# Dysfunction, activity limitations, participation restriction and contextual factors in South African women with pelvic organ prolapse

**DOI:** 10.4102/sajp.v75i1.933

**Published:** 2019-02-28

**Authors:** Corlia Brandt, Elizabeth C. Janse van Vuuren

**Affiliations:** 1 Department of Physiotherapy, University of the Witwatersrand, South Africa; 2 Faculty of Business and Economic Science, University of the Free State, South Africa

## Abstract

**Background:**

Pelvic organ prolapse (POP) is a multifactorial, poorly understood condition impacting quality of life (QOL). The pathology and aetiology might imply population-specific differences in domains of the International Classification of Function, Disability and Health (ICF). There is, however, a lack of research in this regard in South Africa.

**Objectives:**

To describe the dysfunction, activity limitations, participation restrictions and contextual factors in South African women with POP.

**Method:**

One hundred women were conveniently sampled in a primary health care setting. They completed a self-compiled medical and exercise history questionnaire, the standardised Prolapse-Quality of Life (P-QOL) questionnaire and the Visual Faces Scale. The stage of prolapse was determined by the Pelvic Organ Prolapse Quantification (POP-Q) Scale. Means, medians, standard deviations, percentages and frequencies were calculated.

**Results:**

Eighty-six per cent had a stage III POP, 57% had overactive bladder, 50% had constipation, 37% had stress urinary incontinence, 31% had urge urinary incontinence, 32% had incomplete emptying and 30% had anal incontinence. Comorbidities included cardiovascular disease (65%), depressive symptoms (12%) and hypothyroidism (18%). Other contextual factors included limited physical activity (80%), an increased body mass index (29 kg/m^2^), older age (59 years) and unemployment (80%). Quality of life was affected in the severity, social, emotional and sleep/energy domains (median scores were 66.7% – 33.3%).

**Conclusion:**

The dysfunction domain of the ICF was similar to other populations with POP. Activity and participation restrictions included social, emotional and sleep/energy aspects. Contextual factors seem to be population-specific, possibly leading to differences comparing QOL amongst different populations.

**Clinical implications:**

Activity and participation restrictions, as well as contextual factors, may differ in different populations with POP. Interactions between contextual factors and movement impairment should be considered during management and be further investigated.

## Introduction

Pelvic organ prolapse (POP) can be defined as a multifactorial condition which presents as the downward displacement of the pelvic organs from their anatomical position and is associated with bladder and/or bowel dysfunction, and/or sexual dysfunction. Pelvic organ prolapse occurs in approximately 46% – 73% of women in South Africa (Cronje 2011). Increasing evidence shows that women with POP seek treatment primarily to improve their quality of life (QOL) (Srikrishna et al. 2008). Although it is a debilitating condition, patients are reluctant to talk about their problems and many times have symptoms for years. Women adapt their lifestyle and physical activities to the symptoms they experience, which in turn can have a negative effect on their health (Bo et al. 2015).

The International Classification of Functioning, Disability and Health (ICF) classifies health and health-related states. According to this system, the causes of the dysfunctional pelvic floor are classified as the pathophysiological component of the ICF. The dysfunctional pelvic floor muscles manifest the impairment, while the symptoms the patients experience owing to this dysfunction are classified as the disability. Activities and participation restrictions cover domains that denote aspects of capacity and performance (functioning) from an individual and societal perspective (WHO 2002). In the case of POP, the ICF would, therefore, refer to how the symptoms and conditions affect the women’s QOL and participation in functional activities (Bo et al. 2015; WHO 2002). In a survey conducted by Muller (2010), women with self-reported POP in the USA reported issues such as bladder and bowel control, the inability to enjoy sports and physical activities, compromised sexual relationships, the inability to exercise for cardiovascular health and weight management, physical pain and discomfort, and compromises in the workplace, as affecting their QOL. Having a POP may be socially embarrassing and may cause women to avoid certain social situations, such as to withdraw from participating in leisure activities. The withdrawal may lead to a lifelong avoidance of health and fitness activities, a lower activity level and an increase in mortality and morbidity (Bo et al. 2015).

Functioning and disability are also affected by a dynamic interaction between the health condition, such as POP, and contextual factors. Contextual factors include both personal and environmental factors (WHO 2002). Several studies have indicated that contextual factors, such as older age, menopause, body mass index (BMI), birth history and a history of previous surgery, are associated with POP (Weber & Richter 2005). Many of the risk factors for POP and urinary incontinence are also risk factors for the development of lifestyle diseases, such as hypertension and cardiovascular disease. These risk factors include pregnancy and childbirth, deficient connective tissue (varicose veins, hernia, haemorrhoids), hormonal factors, poor diet, smoking, obesity, lack of exercise, ageing and menopause (Bo et al. 2015; Leuzzi & Modena 2011). It could be postulated that the latter diseases may therefore also present in patients with POP, adding to a decreased QOL and lifestyle changes (Leuzzi & Modena 2011).

However, as the context and environment differ in different populations, health care settings and geographical areas, it may be expected that research findings on activity limitations and participation restrictions may not always be generalisable to different populations. Population-specific studies may add to clarifying the lack of understanding regarding the effect POP has on activity limitations and participation restriction, especially in South Africa (Srikrishna et al. 2008).

Population-specific studies on POP can contribute to a conceptual framework of information on personal health care, prevention and promotion by indicating social hindrances in South African women, who may need support and facilitation. Secondly, they may identify bio-psychosocial aspects which may negatively affect movement, leading to identified disability, activity limitations and participation restrictions. Lastly, they may contribute to the development of the current health care system in terms of evaluation and policy formulation regarding women’s health in South Africa (WHO 2002).

The aim of this study was therefore to describe the dysfunction, activity limitations, participation restrictions and contextual factors in South African women with POP.

## Methods

All eligible patients from a population of women attending uro-gynaecology clinics in the public and private primary health care sectors were approached for participation. Women (white, Asian and of mixed race) aged over 18 years and scheduled for corrective surgery for POP were included. Inclusion criteria were based on current aetiological literature. A study by Hoyte et al. (2005) compared the pelvic morphology between asymptomatic African-American women and white nulliparous women. They found significant differences in the mm. levator ani volume, the levator-symphysis gap, bladder neck position, urethral angle and the pubic arch angle. Such differences may lead to differences in the development of pelvic floor dysfunction (Hoyte et al. 2005). This is supported by another study on racial differences in POP that reported the prevalence of symptomatic prolapse to be five times higher for white and Latina women when compared with African-American women (Whitcomb et al. 2009). Populations should, therefore, be carefully selected and the interventions specifically tailored to yield clinically valid results.

Pregnant women, women with stage IV POP and women suffering from systemic neuro-musculoskeletal/psycho-sexual disorders were excluded.

### Measurement

A total of 100 participants completed the standardised Prolapse-Quality of Life Questionnaire (P-QOL) in their language of choice (English or Afrikaans), assisted by a translator when required. The P-QOL assesses urinary, sexual and defecation symptoms, as well as physical, social and emotional aspects (Digesu et al. 2005). The questionnaire is scored out of 100 for each domain, with higher scores indicating poorer QOL. This questionnaire has been shown to have good validity (Cronbach α > 0.80), inter-rater reliability (*p* < 0.1, *r* > 0.5) and test–retest reliability (*r* = 0.872) (Digesu et al. 2005). It has also been validated in a South African population (Brandt, Van Rooyen & Cronje 2016).

The QOL assessment was accompanied by the completion of a questionnaire developed by the first author to gather data on the medical and exercise history of the participant. Demographic information, general health, symptoms and signs, exercise history and gynaecological history – including the stage of POP – were gathered in this questionnaire.

The Pelvic Organ Prolapse Quantification (POP-Q) scale is recommended by the International Continence Society as the standard method of describing the stage of POP in all research studies and was also used in our study. It defines the location of six points (two on the anterior vaginal wall, two in the superior vagina and two on the posterior vaginal wall), with reference to the plane of the hymen. The anatomical position of the points is then measured as the number of centimetres proximal (negative number) or centimetres distal to the hymen (positive number). The stage of POP was assessed by the same gynaecologist in all participants, as described by the International Continence Society (Haylen et al. 2016).

A five-item Likert scale, namely the Visual Faces Scale (VFS), was completed by all participants to assess pelvic and lower back pain. This is a reliable and valid method of pain assessment (with a median validity and test–retest reliability coefficient of 0.82 and 0.70, respectively), which has been used in previous studies on POP (Heit et al. 2002).

### Pilot study

A pilot study on six eligible participants was conducted to test the methodology of the study, following the protocol as described above. The data forms and questionnaires were checked for errors and understanding of instructions. No changes were made following the pilot study and the data collected were included for analysis.

### Data analysis

Data analysis was performed by an independent biostatistician and summarised using descriptive statistics. Analysis included calculation of frequencies and percentages for categorical data, and means, standard deviations, minimum and maximum values in the case of a symmetric distribution of the continuous variables. Where data were skewed positively or negatively, the median, 25th percentile and 75th percentile were also included for interpretation.

### Ethical considerations

This descriptive study was approved by the Ethics Committee of the Faculty of Health Sciences of the University of the Free State, South Africa (ECUFS no. 25/2012). Written, informed consent from the participants and permission from the institutions where the study was conducted were obtained.

## Results

The results for demographic data are summarised in Table 1. Participants had a mean age of 59 years (standard deviation [SD] = 9.13), a median BMI of 28.67 kg/m^2^ (interquartile range [IQ] 26.08–32.99) and did manual labour (60%). Gynaecological history included information regarding the number of pregnancies (mean = 3.3, SD = 1.58) and deliveries (median = 3, IQ range = 2–3) (Table 1). Interestingly, a relatively small percentage of participants were using hormone replacement therapy (17%), considering the number of women who were in their menopause (31%) or who were post-menopausal (54%).

**TABLE 1:** Results for demographic variables (continuous).

Variable	*n*	Skewness	Mean	SD	Min	Max	Median	25th percentile	75th percentile
Age (years)	98	−0.706	59.00	9.131	29	75	60	n/a	n/a
Weight (kg)	97	1.245	79.40	18.563	50	149	75	66	86
Length (cm)	100	−1.417	162.27	9.846	106	195	162	159	167
Pregnancies	99	0.829	3.303	1.581	0	8	3	2	4
Deliveries	99	1.063	2.97	1.417	0	8	3	2	3

Min, minimum; Max, maximum; SD, standard deviation; n/a, not applicable; *n*, sample size.

**FIGURE 1:**
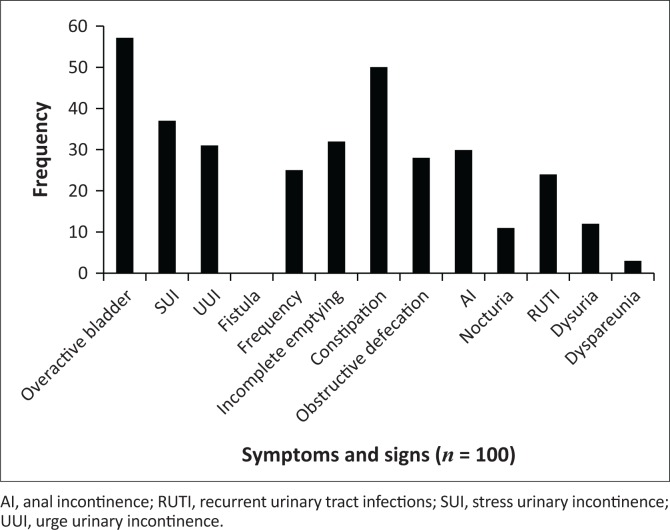
Symptoms and signs.

The participants presented with common symptoms and signs associated with POP and pelvic floor muscle dysfunction (Figure 1). A total of 86% (*n* = 100) of the participants had a stage III POP. Most participants complained of an overactive bladder (57%), followed by complaints of constipation (50%), stress urinary incontinence (37%), urge urinary incontinence (31%), incomplete emptying (32%) and anal incontinence (30%). The other symptoms and signs with a lesser frequency of occurrence are depicted in Figure 1.

Of note is the fact that assessment of pain by the VFS found that the participants experienced a median value of zero pelvic pain (IQ range 2) and a mean intensity value of 1.49 lower back pain (SD = 1.654).

Results of the P-QOL questionnaire are depicted as the median values for each domain owing to the non-parametric distribution of data. These values are depicted in Table 2, together with a summary of the evidence-based values found by previous studies for symptomatic women diagnosed with POP, to indicate the cut-off values regarding classification as symptomatic or non-symptomatic. The general health perception of most of the participants ranged from good (34% of participants) to fair (47% of participants). These findings did not indicate impaired QOL when compared to the evidence on symptomatic scores. However, the median value of 25% calculated for the general health item was on the border of symptomatic classification (namely 25% – 70%). Contrary to the general health domain, the prolapse impact score of 71% of the participants, ranging from 67% to 100%, indicated impairment in this domain.

**TABLE 2:** Table of median domain scores of the prolapse-quality of life questionnaire preoperatively, *n* = 100.

Domain	Median value (%) and IQ range (*n* = 100)	Evidence-based symptomatic values (Median % scores)
Prolapse impact	66.67 (33.33–100.0)	57–100
Role limitations	33.30 (0.00–75.0)	67
Physical limitations	33.30 (0.00–75.0)	50–67
Social limitations	33.30 (0.00–66.67)	16–56
Personal relationships	0.00 (0.00–66.67)	50–67
Emotions	44.40 (11.1–72.2)	44–67
Sleep/energy	33.30 (16.67–50.0)	33–67
Severity measures	25.00 (12.5–41.67)	11–42

IQ, interquartile range.

Other domains that were found to be impaired were social limitations, emotional aspects, sleep/energy and severity measures (Table 2). It seems that the domains that included physical activity as a limitation (namely the domains of role and physical limitations) were less impaired by the prolapse. Personal relationships were least affected with a median value of 0% (IQ range = 0–66.67).

Activity limitations, participation restriction and contextual factors were further described by the results of the self-compiled questionnaire. The participants’ lifestyles were characterised by low levels of sport participation (15% of participants) and a lack of exercise. Only 21% of participants did cardiovascular exercise, which was mostly walking in 18% of the cases. Fifteen per cent of the women had been introduced to pelvic floor muscle exercises, while only 7% were familiar with exercises to strengthen the core muscles (Table 3).

**TABLE 3:** Results for demographic variables (categorical), *n* = 100.

Variable	Frequency	Percentage
**Language**		
Afrikaans	95	95
English	5	5
**Work**		
Manual labour	60	**60**
Office work	20	20
Pensioner	20	20
**Participation in sport**		
Yes	15	15
No	85	**85**
**Type of exercise activities**		
Jogging	1	1
Swimming	2	2
Tennis	0	0
Walking	18	**18**
Weight training	1	1
Pilates/yoga	1	1
Line dance	1	1
Fishing	2	2
**Level of participation**		
Social	24	24
Provincial	0	0
National	0	0
**Comorbidities**		
Heart disease	14	**14**
Vascular disease	17	**17**
Pulmonary disease	3	3
Cancer	1	1
Allergies	21	21
Previous surgery	56	**56**
Inflammatory disease	19	19
Diabetes mellitus	3	3
Hypothyroidism	3	3
Depression	1	1
Psoriasis	1	1
**Medication**		
Hypertension/angina	47	**47**
Hormone replacement therapy	17	**17**
Anti-inflammatory medication	8	8
Antidepressants	12	**12**
Hypothyroidism	18	**18**
Vitamins and minerals	9	9
Gastric ulcer	3	3
Overactive bladder	2	2
Cholesterol	18	**18**
Pain	7	7
Diabetes mellitus	9	9
Asthma	7	7
Constipation	1	1
Insomnia	3	3
Anticoagulant	5	5
Antihistamines	2	2
Malaria	1	1
**Smoking**		
Yes	20	20
No	80	80
**History of pelvic floor muscle exercise**		
Yes	15	15
No	85	**85**
**History of core/stability exercise**		
Yes	7	7
No	93	**93**
**Menopausal state**		
Pre-menopause	15	15
Peri-menopause	31	31
Post-menopause	54	54
**History of pelvic/abdominal surgery**		
Yes	45	45
No	55	55
**Type of surgery**		
Anterior repair	25	25
Posterior repair	5	5
Partial colonostomy	1	1
Hysterectomy	18	18
Appendectomy	9	9
Laparoscopy	2	2
Laparotomy	3	3
Gall bladder	1	1
Hernia repair	2	2
Trans-obturator tape (TOT) sling	1	1

Note: Bold results indicate important findings referred to in the text.

The results on the lack of exercise could also be informed by the fact that many of the participants had heart and/or vascular disease (14% and 17% of participants, respectively). Forty-seven per cent was taking medication for hypertension and 18% for cholesterol. Hypothyroidism was also an area of concern (18% of participants used medication), (Table 3).

A cross-tabulation of exercise participation and heart disease indicated that 31% of the participants who were physically inactive (*n* = 95) had heart disease, compared to the 13% of participants who were physically active (*n* = 15). A cross-tabulation between exercise participation and the use of antidepressants indicated that none of the participants who did exercise (*n* = 15) used antidepressants. Fourteen per cent of the participants who did not exercise (*n* = 85) used antidepressants.

## Discussion

**FIGURE 2:**
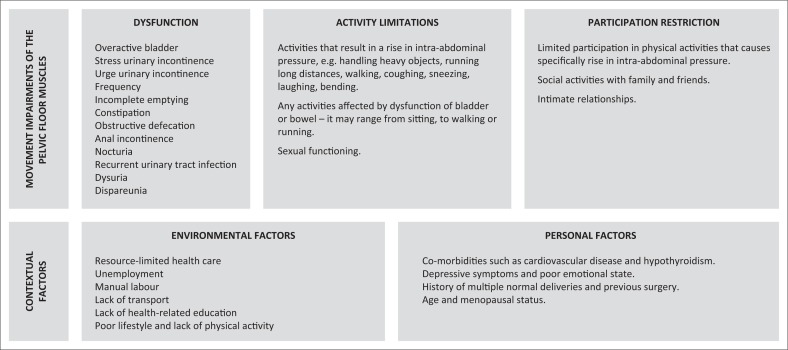
Discussion of findings using an International Classification of Function, and Disability and Health framework for patients with pelvic organ prolapse in a South African context.

According to the ICF, the dysfunction of the pelvic floor muscles (such as reduced force, timing of contraction and endurance) can be classified as the impairment component. The symptoms owing to the pelvic floor dysfunction (such as urinary incontinence, faecal incontinence and POP) can be defined as the dysfunction according to the ICF. The activity or participation restriction is the effect that the symptoms or condition has on the women’s QOL and participation in fitness activities (Bo et al. 2015). Related to this framework, the participants in this study were characterised by the presence of overlapping risk factors (the personal and environmental factors) for POP and lifestyle diseases, disability in the form of symptoms and signs owing to pelvic floor dysfunction and activity and participation restrictions (social, emotional and physical components) (Figure 2).

### The dysfunction domain

The participants presented with common symptoms and signs associated with POP and pelvic floor muscle dysfunction. Bladder and bowel symptoms, as well as a bulging feeling, were the prominent symptoms affecting the participants preoperatively, and not symptoms such as pain (Bo et al. 2015). It was interesting to note that although most participants complained of an overactive bladder and constipation, these were the least frequent conditions for which medication was taken. Only two participants (*n* = 100) reported taking medication for an overactive bladder, while one participant (*n* = 100) reported taking medication for constipation (Table 3). The limited pharmacological management may be explained by the fact that most of the participants presented to the clinic for the first time during the study, therefore had not been assessed or managed before. Coinciding with the most common bladder and bowel symptoms reported by the participants (namely overactive bladder and constipation) were the results regarding the type and stage of POP. Commonly associated with cystocele and rectocele are overactive bladder, constipation and incomplete emptying (De Boer et al. 2010). The presence of multiple associated symptoms of the bladder and bowel has been noted in studies on the pathophysiology and aetiology of POP, indicating the multifactorial pathophysiology and complexity of the disease leading to a decreased QOL (Haylen et al. 2016; Weber & Richter 2005).

### The participation and activity restrictions

The QOL domains that were most affected according to the P-QOL were the impact and severity of the prolapse, as well as the emotional, social and sleep/energy domains. Many previous studies have found mostly prolapse impact to be affected. However, in these studies, emotional, social and sleep/energy domains were less affected when compared to the scores of physical, personal and role limitations (Cam et al. 2007; Digesu et al. 2005). Our sample did, however, report a lack of physical activity and exercise participation in the demographic questionnaire but did not report the level of physical activity as a QOL restriction according to the P-QOL results. The fact that limited physical activity was not indicated as a QOL restriction may suggest that the lack of physical activity should rather be seen as a contextual factor in our study, which could be part of a larger lifestyle issue.

### Contextual factors (personal and environmental)

A reason for the difference in the findings of the affected QOL domains, when compared to the available international literature, might be explained in terms of the difference in personal, environmental, social, educational and economic factors interacting with the physical/emotional well-being in the different populations (Eleje et al. 2015). All the mentioned studies were conducted in developed world countries where resources and social, educational and economic factors might have less of an impact on patients and how they feel when compared to a resource-restricted health system from which our participants were mostly recruited (Eleje et al. 2015). The socio-economic and general health circumstances in our study were perhaps demonstrated by the fact that most participants were either unemployed or pensioners, with the median of their general health domain results being on the border of the symptomatic classification.

Most previous studies found general health not to be an affected domain (median ranges from 25% to 50%), except a study by Cam et al. (2007) which found a median score of 75% in the general health domain (Cam et al. 2007; Claerhout et al. 2010; De Oliveira, Tamanini & de Aguiar Cavalcanti 2009; Lenz et al. 2009; Manchana & Bunyavejchevin 2010; Svihrova et al. 2014). Their study did not differ with regard to methodology or demographic variables from the others. The results of our study with regard to the general health domain were relatively low, although on the border of symptomatic classification when compared to normal values. Moderate scores were obtained in the general health domain despite the fact that many participants had comorbidities that could potentially affect their general health (Table 3). The presence of comorbidities, therefore, did not seem to affect their impression of their general health extensively. Most previous studies also found a high score in the prolapse impact domain (median ranges from 57% to 100%). It could, therefore, be postulated that these findings might be an indication of the sensitivity of the P-QOL, where findings can be contributed to the prolapse impact rather than poor general health.

Other personal factors, such as older age, menopause, BMI, birth history and a history of previous surgery, have been indicated to be associated with POP (Fialkow et al. 2002). The statement by Fialkow et al. (2002) may be supported by similar prevalence and demographic findings in our study and other studies conducted on patients with pelvic floor dysfunction (Frawley et al. 2010; Jarvis et al. 2005; Pauls et al. 2013; Vakili et al. 2005). Most women had a parity of two to three normal deliveries, and a half or more of the sample in all studies had a history of previous hysterectomy or reconstructive surgery, compared to the 45% (*n* = 100) found in our study. The studies also reported a BMI in the range of 27kg/m^2^. This BMI is slightly higher as reported in our study (median of 29kg/m^2^). The mean age in all studies was approximately 60 years (compared to the mean of 59 years in our sample), which explains why the majority of the samples were classified as post-menopausal.

Data from previous studies indicate that approximately 50% of post-menopausal women have hypertension or are taking anti-hypertensive therapy. This high prevalence rate was reflected in our results where 47% of our participants were receiving medication for hypertension and 31% had a history of cardiovascular disease. The importance of these findings is that hypertension has been labelled as one of the major cardiovascular risk factors in post-menopausal women (Leuzzi & Modena 2011).

The pathophysiology of post-menopausal hypertension related to the post-menopausal stage can firstly be explained by the lack of oestrogen during this period. However, it was interesting to note that only 17% of our sample was receiving hormone replacement therapy at the time of the study. The second cause of hypertension in post-menopausal women can be endothelial dysfunction, while obesity is the third factor that could contribute to the development of hypertension in this specific group of patients (Leuzzi & Modena 2011). This explanation could relate to the increased BMI (28.67 kg/m^2^), as well as the prevalence of hypertension/cardiovascular disease, which was found in our sample.

Savoy and Penckofer (2015) have also reported a link between increased cardiovascular morbidity and mortality and depressive symptoms, especially in women. More than 15% of patients with cardiovascular disease have depressive symptoms, while women are twice as likely to have these symptoms compared to men. Raising the concern, however, is the fact that depressive symptoms, as identified in our sample, are also defined as independent risk factors for cardiovascular disease and are often under-diagnosed. It is the adverse effect of depressive symptoms, even if not clinically diagnosed, on the compliance to healthy lifestyle behaviour changes that contributes to the risk of developing cardiovascular disease and potentially other diseases, such as POP. Approximately 70% of the participants in our sample who had depression/were treated for depressive symptoms (*n* = 13) also had cardiovascular disease. None of these participants did any form of exercise which reflects upon the above statement regarding the compliance to healthy lifestyle behaviour changes in patients with depressive symptoms. These participants’ BMI was also higher (e.g. 33.23 kg/m^2^) than the average of the whole sample (28.67 kg/m^2^). However, it must be considered that an increased BMI, as well as a lack of exercise, are also independent risk factors for cardiovascular disease.

Furthermore, 63% of the 19 participants in our study who had hypothyroidism also had cardiovascular disease or were treated for the same. Subclinical or mild hypothyroidism occurs in approximately 5% – 20% of post-menopausal women and is associated with cardiac disease, and is often seen in patients with POP. However, in a large case-cohort study comprising 736 cases that had a myocardial infarction and a sub-cohort of 2927 members, no significant association was found between myocardial infarction and subclinical hypothyroidism in post-menopausal women. The authors suggested that the inclusion of women with higher thyroid-stimulating hormone levels and who were younger might yield different results (LeGrys et al. 2013).

To conclude, a comparison of the findings of our study to others indicated that the sample presented with similar dysfunction when compared to other populations with POP. However, differences were noted in the QOL domains that were affected (activity and participation restrictions), as well as in the contextual factors that were present. The findings, therefore, support the hypothesis that activity limitations and participation restrictions might be population-specific. Differences in findings between populations could be owing to specific differences in contextual factors interacting with the other domains of the ICF.

## Recommendations

Based on the findings of this study, recommendations could be suggested for management strategies. The presence of comorbidities, such as cardiovascular disease, depressive symptoms, hypothyroidism and the lack of physical activity, might suggest addressing dietary and lifestyle changes in these patients, with or without drug therapy. Changing lifestyle behaviour in these patients could be informed by the fact that patients with POP also adapt their lifestyle and physical activities owing to the symptoms they experience, for example, patients with overactive bladders or urge urinary incontinence may start limiting their physical activity for fear of leaking (Kozica et al. 2012). Lifestyle changes would include aspects such as weight control, increased physical activity, alcohol moderation, decreased sodium intake, increased consumption of fresh fruit and vegetables and low-fat dairy products (Leuzzi & Modena 2011). These lifestyle changes are very similar to those that are proposed for women with POP – owing to the overlap of aetiological and risk factors between these diseases and could, therefore, help to manage both diseases as well as the possible interaction between them.

Increasing physical activity would not only help to lower depressive symptoms but also to prevent the development of cardiovascular disease. The suggestion to promote increasing physical activity in these patients is supported by the cross-tabulations in the study, which indicated that patients who were doing physical activity might be less likely to have cardiovascular disease or depressive symptoms. Bernard et al. (2015) conducted a randomised controlled trial in post-menopausal women investigating the effect of a 6-month walking intervention programme on depressive symptoms in this population. They found that three sessions per week of moderate-intensity walking (supervised and home-based) decreased depressive symptoms in their sample, even with a minimal adherence of 50% to their programme.

The minimal adherence that the study of Bernard et al. (2015) found could be explained by the study by Savoy and Penckofer (2015). They found that subsyndromal depressive symptoms in women are significantly inversely related to health-promoting lifestyle behaviours and general QOL. Depressive symptoms interfere, especially with compliance to alter lifestyle behaviour and other treatment plans. Feelings of tiredness, hypersomnia and the consequence of insomnia would influence the physical capability of the participant to execute exercises, in other words, the input and output mechanisms related to sensorimotor control would be influenced by fatigue. On a cognitive (processing) level, it would also have an effect on concentration and thinking (Jull et al. 2015). Future research should therefore also focus on the effect that the contextual factors may have on the impairments (such as sensory-motor control aspects of the pelvic floor muscles) and compliance to exercise programmes.

It can also be suggested that the study be repeated in different regions of South Africa, as cultural differences (therefore differences in contextual factors) may perhaps lead to differences in activity and participation restrictions even within a South African context.

## Conclusion

This study described the dysfunction (symptoms and signs of pelvic floor dysfunction) with which South African women with POP might present. This domain of the ICF seems similar to studies conducted in other populations with POP. The most prevalent activity and participation restrictions (QOL domains) that were affected were social, emotional and sleep/energy domains. This differed from studies in other populations which reported physical and role limitations to be the most affected. Lastly, several contextual factors (which seem to be population-specific) were identified that might interact with the domains of the ICF, leading to differences in these domains when comparing different populations with POP. Future research should focus on the effect of lifestyle interventions and interactions between contextual factors and the impairment domains of the ICF in women with POP, in order to determine the best management strategies.
